# Isolation and Characterization of Novel Lytic Bacteriophages Infecting Epidemic Carbapenem-Resistant *Klebsiella pneumoniae* Strains

**DOI:** 10.3389/fmicb.2020.01554

**Published:** 2020-07-21

**Authors:** Min Li, Min Guo, Long Chen, Chaowang Zhu, Yuyi Xiao, Pei Li, Hongxiong Guo, Liang Chen, Wei Zhang, Hong Du

**Affiliations:** ^1^College of Veterinary Medicine, Nanjing Agricultural University, Nanjing, China; ^2^Key Lab of Animal Bacteriology, Ministry of Agriculture, Nanjing, China; ^3^MOE Joint International Research Laboratory of Animal Health and Food Safety, College of Veterinary Medicine, Nanjing Agricultural University, Nanjing, China; ^4^Department of Clinical Laboratory, The Second Affiliated Hospital of Soochow University, Suzhou, China; ^5^Department of Clinical Laboratory, Zhangjiagang Hospital Affiliated to Soochow University, Zhangjiagang, China; ^6^Department of Clinical Laboratory, The North District of Suzhou Municipal Hospital, Nanjing Medical University, Suzhou, China; ^7^Department of Expanded Program on Immunization, Jiangsu Provincial Center for Disease Control and Prevention, Nanjing, China; ^8^Center for Discovery and Innovation, Hackensack Meridian Health, Nutley, NJ, United States; ^9^Hackensack Meridian School of Medicine, Seton Hall University, Nutley, NJ, United States

**Keywords:** bacteriophages, carbapenem-resistant, host range, *Klebsiella pneumoniae*, sewage

## Abstract

Carbapenem-resistant *Klebsiella pneumoniae* (CRKP) poses a significant clinical problem given the lack of therapeutic options available. Alternative antibacterial agents, such as bacteriophages, can be used as a valuable tool to treat the infections caused by these highly resistant bacteria. In this study, we isolated 54 phages from medical and domestic sewage wastewater between July and September 2019 and determined their host ranges against 54 clinical CRKP isolates, collected from a tertiary hospital in eastern China. The 54 CRKP isolates were from 7 sequence types (STs) and belonged to 9 capsular K locus types, harboring *bla*_KPC–__2_ (*n* = 49), *bla*_NDM–__1_ (*n* = 5), and *bla*_IMP–__4_ (*n* = 3). Among them, the epidemic KPC-2-producing ST11 strains were most predominant (88.9%). The 54 phages showed different host ranges from 7 to 52 CRKP isolates. The total host ranges of three phages can potentially cover all 54 CRKP isolates. Among the 54 phages, phage P545, classified as a member of Myoviridaes, order Caudovirales, had a relatively wide host range (96.3%), a short latent period of 20 min, and a medium burst size of 82 PFU/cell and was stably maintained at different pH values (4–10) and temperatures (up to 60°C). P545 showed the ability to inhibit biofilm formation and to degrade the mature biofilms. Taken together, the results of our study showed that the newly isolated phage P545 had a relatively wide host range, excellent properties, and antibacterial activity as well as antibiofilm activity against a clinical CRKP ST11 isolate, providing a promising candidate for future phage therapy applications.

## Introduction

Carbapenem-resistant *Klebsiella pneumoniae* (CRKP) has spread globally and become a significant clinical threat because of the lack of therapeutic options available. The carbapenem-resistant genes were mostly plasmid borne, thereby facilitating the horizontal transfer of resistance between different species and strains. Worrisome is that CRKP strains have recently developed resistance to some last-line antibiotics, such as polymyxin and tigecycline (Zhang et al., 2018). Although some novel β-lactam/β-lactamase inhibitor combinations, e.g., cefatazidime/avibactam, meropenem/vaborbactam, and imipenem/relebactam, were approved for the treatment of CRKP infections, they were not effective against all carbapenemase producers. Consequently, antibiotic therapeutic choices against CRKP remain limited, while other measures, such as phage therapy, have been considered as alternative measures to prevent and control CRKP infections (Ciacci et al., 2018; Anand et al., 2019).

Bacteriophages, also known as phages, are viruses that can invade and replicate in bacterial cells, and for the case of lytic phages, they are able to disrupt bacterial metabolism and cause the bacteria to lyse. Phages were discovered and clinically implemented against bacterial infections approximately 100 years ago, even before the discovery of antibiotics (Kwan et al., 2005). However, phage therapy was gradually ignored along with the wide application of antibiotics (Criscuolo et al., 2017; Lazaro-Perona et al., 2018). The progressive reduction of the effectiveness of antibiotics has recently generated renewed interest in revisiting phage therapy.

During clinical practice, phages can be used systematically through oral or injection administration, or locally on wounds or burns, as well as in the hospital environment and on medical devices. Phage therapy has been used against some Enterobacteriaceae pathogens, and among them, *Escherichia coli* phages (Chen et al., 2017; Cieplak et al., 2018) and *Salmonella* phages (Huang et al., 2018) were the most studied and applied. *K. pneumoniae* phages also had some applications, such as in burns (Chadha et al., 2017) and diabetes-related foot ulcers (Morozova et al., 2018). However, studies on phages against clinical CRKP strains were still limited. In the study reported here, we isolated 54 phages from domestic and hospital sewage wastewater and examined their activities against 54 CRKP strains collected from a tertiary hospital in eastern China, within one of the CRKP endemic regions.

## Materials and Methods

### Bacteria and Growth Conditions

A total of 54 unique (one isolate per patient) CRKP isolates, collected from blood (*n* = 7), abdominal cavity (*n* = 5), respiratory tract (*n* = 35), urinary tract (*n* = 4), and other body sites (*n* = 3), from a tertiary hospital in eastern China between 2016 and 2018 were used as host bacteria to determine the host ranges of phages. Details of the bacterial isolates used in this study are listed in Table 1. All isolates were routinely cultured in Luria*–*Bertani (LB) broth at 37°C on an orbital shaker at 180 rpm. Phosphate-buffered saline (0.1 M Na_2_HPO_4_, 0.15 M NaCl_2_, pH 7.2) was used for dilution and wash of *K. pneumoniae* cultures.

**TABLE 1:** Molecular characteristics of the 54 *K. pneumoniae* clinical isolates.

**Sequence types (STs)**	**Number (%)**	**K locus (KL) types (*n*,%)**	**Carbapenemases (*n*,%)**	**ESBLs and AmpC (*n*,%)**	**Sources (*n*,%)**
ST11	48 (88.9)	KL64 (24, 50.0) KL47 (23, 47.9) KL25 (1, 2.1)	KPC-2 (48, 100.0) NDM-1 (2, 4.2)	CTX-M-65 (43, 89.6) CTX-M-14 (1, 2.1) SHV-12 (16, 33.3) DHA-1 (10, 20.8)	Blood (7, 13.0) Abdominal cavity (5, 9.3) Respiratory tract (30, 55.6) Urinary tract (4, 7.4) Others (2, 3.7)
ST147	1 (1.9)	KL107 (1, 100.0)	KPC-2 (1, 100.0)	−	Respiratory tract (1, 1.9)
ST230	1 (1.9)	KL136 (1, 100.0)	IMP-4 (1, 100.0)	CTX-M-3 (1, 100.0)	Respiratory tract (1, 1.9)
ST3155	1 (1.9)	KL139 (1, 100.0)	IMP-4 (1, 100.0)	−	Others (1, 1.9)
ST3369	1 (1.9)	KL116 (1, 100.0)	NDM-1 (1, 100.0)	−	Respiratory tract (1, 1.9)
ST367	1 (1.9)	KL1 (1, 100.0)	IMP-4 (1, 100.0) NDM-1 (1, 100.0)	CTX-M-55 (1, 100.0)	Respiratory tract (1, 1.9)
ST656	1 (1.9)	KL14 (1, 100.0)	NDM-1 (1, 100.0)	CTX-M-3 (1, 100.0)	Respiratory tract (1)

*−, negative.*

### Antimicrobial Susceptibility Testing

Antimicrobial susceptibility testing was conducted using the standard broth microdilution method and followed the Clinical & Laboratory Standards Institute (CLSI) guidelines. Meropenem and imipenem were tested from 0.25 to 32 μg/mL. The 2018 CLSI breakpoints were used to interpret the susceptibility results. This experiment was performed in three biological replicates. *Escherichia coli* ATCC^®^25922 was used as a negative control.

### Genetic Background of CRKP Strains

Multilocus sequence typing (MLST) was used to investigate the sequence types (STs) of the total 54 CRKP isolates. In brief, polymerase chain reaction (PCR) detection followed by Sanger sequencing was used to examine seven conserved housekeeping genes including *gapA*, *infB*, *mdh*, *pgi*, *phoE*, *rpoB*, and *tonB* (Diancourt et al., 2005). Allelic profiles and sequence types were determined based on the *Klebsiella* locus/sequence definitions database^1^. Capsular types were determined by *wzi* sequencing (Brisse et al., 2013) and the K locus types (KLs) were inferred using the same *Klebsiella* locus/sequence definitions database as described above. In addition, ST11 KL64 and KL47 capsular types were examined by a multiplex PCR scheme described previously (Yu et al., 2018).

### Detection of Carbapenemases, Extended-Spectrum Beta-Lactamases, and AmpC Genes

Polymerase chain reaction was performed to investigate the presence of carbapenemase-encoding genes, including *bla*_KPC_, *bla*_NDM_, *bla*_VIM_, *bla*_IMP_, and *bla*_OXA–__48_-like. In addition, we examined the extended-spectrum beta-lactamases (ESBLs) (CTX-M, SHV, and TEM) and AmpC cephalosporinases (CMY, ACT, and DHA) using PCR followed by Sanger sequencing. Oligonucleotide primers used for screening the aforementioned genes were described in previous reports (Mammeri et al., 2010; Bokaeian et al., 2015; Candan and Aksoz, 2015).

### Isolation of Phages

Phages were isolated from the filtered (0.22-μm pore size) sewage, including medical sewage from three sewage treatment stations of local hospitals (site A, B, C) and domestic sewage from a local sewage treatment plant (site D) (Supplementary Table S1).

The purification, counting, and propagation of phages were performed using the double-layer agar plate method (Adams, 1959). SM buffer (50 mM Tris−HCl [pH 7.5], 100 mM NaCl, 10 mM MgSO_4_, and 0.01% gelatin) was used for the dilution of the phages. Phage suspensions were centrifuged at 5000 × *g*, 4°C for 10 min, and then the supernatants were filtered through 0.22-μm filters (Merck Millipore, Germany) and stored at either 4°C or at -80°C in glycerol (3:1 [v/v]) (Gu et al., 2011). All the sterile operations were carried out in class II biosecurity cabinets with institutional safety approval.

### Host Range Determination of Phages

The host ranges of the phages were determined against 54 CRKP strains using spot testing (Chen et al., 2017). Briefly, 10 μL of the purified phage suspensions (∼10^9^ PFU/mL) were spotted onto freshly seeded lawns of the isolates and left to dry before incubation for 6 h at 37°C (Kutter, 2009). Host bacteria were determined by the observation of lytic spots. The host-range frequencies were analyzed using the GraphPad Prism 5 software package. Host ranges of 54 phages were illustrated by HemI software (Deng et al., 2014).

### Examination of Bacteriophage Morphology by Electron Microscopy

We selected three phages−P545, P539, and P507−for morphological analysis by transmission electron microscopy (TEM), as they had different host ranges and plaque morphology. The filtrates of phages P545, P539, and P507 were applied to a copper grid, respectively, before negative staining with phosphotungstic acid (PTA, 2% w/v). Electron micrographs were observed using an H_7650 transmission electron microscope (Hitachi, Tokyo, Japan). Bacteriophage morphology and taxonomy were confirmed following the guidelines from the International Committee on Taxonomy of Viruses^2^.

### Optimal Multiplicity of Infection

A relatively wide host-range phage, P545, identified from the current study was further selected to determine its basic characteristics, including optimal multiplicity of infection, one-step growth curve, and thermo and pH stability.

Multiplicity of infection (MOI) refers to the ratio of phages to host bacteria during the processes for infection (Ji et al., 2019). *K. pneumoniae* ST11 strain KP4 was used as the host strain and was grown to logarithmic phase at a final concentration of approximately 2 × 10^8^ CFU/mL (OD_600_ ∼0.4). Phage P545 suspensions were then added at different MOIs (phage/bacteria = 10, 1, 0.1, 0.01, 0.001, 0.0001, 0.00001, and 0.000001), and the mixtures were incubated at 37°C for 10 h. The phage titers were then determined immediately by plaque assay after serial dilution (Gong et al., 2016). All assays were conducted in triplicate. The MOI that generated the highest phage titer within 10 h was considered as the optimal MOI.

### One-Step Growth Curve of Phage P545

Bacteriophage latent time and burst size were determined by one-step growth curve as previously described (Pajunen et al., 2000). Phage P545 was added to the exponential-phase KP4 culture (∼2 × 10^8^ CFU/mL) at an MOI of 0.1, followed by adsorption at 37°C for 10 min. The precipitated KP4 strains were then resuspended in 10 mL of fresh LB medium after discarding unabsorbed phages by centrifugation at 5000 × *g* for 10 min at 4°C, followed by incubation at 37°C with shaking at 180 rpm. Samples were taken out at 5-min intervals for 120 min, and a 1% final concentration of chloroform was added to each subsample to release the intracellular phages for determining phage titers using the double-layer agar plate method. All assays were conducted in triplicate.

### Thermo and pH Stability

Thermo and pH stability tests of phage P545 were conducted following a previously described protocol with minor modifications (Laemmli, 1970). In brief, 100 μL of phage was suspended in 900 μL of SM buffer (pH 7.5) and kept at different temperature (i.e., 4, 25, 37, 50, 60, 70, and 80°C) for 1 h. Phage survival rates were determined by the double-layer agar plate method described above. All assays were conducted in triplicate.

To determine pH stability of P545, 100 μL of phage suspension was added to 900 μL of SM buffer with a gradient of pH (range from 1 to 12), followed by incubation at 37°C for 1 h. SM buffer with a gradient of pH (pH 1–12) was adjusted by HCl and NaOH solution, and measured by a pH meter. Similarly, all the samples were diluted and tested immediately by the double-layer agar plate method. All assays were conducted in triplicate.

### DNA Extraction and Genome Sequencing

The phage P545 suspensions were concentrated and purified for genomic DNA extraction as previously described (Sambrook and Russell, 2006). The phage genome was sequenced using the Illumina HiSeq system (Illumina, San Diego, CA, United States). Sequencing reads were *de novo* assembled using Spades 3.11.1 (Bankevich et al., 2012). The whole-genome sequence of phage P545 was deposited in the GenBank database under accession number MN781108. Annotation of the phage genome was conducted by the RAST server (Aziz et al., 2008), followed by manual curation. Phages closely related to P545 were examined using online BLASTn against the NCBI database^3^. Genome alignment of the phages was illustrated using EasyFig.

### Bacteriophage Potency Against Planktonic Cells and Biofilm

To assess the inhibition effectiveness of phage P545 against KP4 planktonic cells, phages were added to the KP4 cultures (OD 0.1, ∼5 × 10^7^CFU/mL) at MOIs of 0.1, 0.01, and 0.001, followed by incubation at 37°C with shaking at 180 rpm. Bacterial cultures with no phages was used as an untreated group. The OD_600_ values of the cultures were determined at 5-min intervals for 70 min. The inhibition effectiveness of P545 were analyzed by the growth kinetics of host bacteria.

To assess the inhibition effectiveness of phage P545 in biofilm formation, 100 μL of exponential-phase bacteria (OD 0.4, ∼2 × 10^8^CFU/mL), 20 μL of phages (at MOIs of 0.1, 0.01, and 0.001), and 80 μL of fresh tryptic soy broth (TSB) medium (Merck, Darmstadt, Germany) were added into each well of 96-well flat-bottomed polystyrene microtiter plates (Sigma-Aldrich, St. Louis, MO, United States) as phage-treated groups. The untreated group consisted of 100 μL of bacteria mixed with 100 μL of fresh TSB medium, while 200 μL of fresh TSB medium was used as the negative control group. All plates were incubated for 24 h at 37°C without shaking. The biofilms were stained with 1% crystal violet as described previously (Wu Y. et al., 2019). Optical density values at 595nm (OD_595__)_ were measured using an ELISA microplate reader (Biotek, Winooski, VT, United States). Each experiment was performed in triplicate.

The degradation activity of P545 against the established mature biofilms of KP4 was evaluated using previously described protocols with minor modification (Cerca et al., 2005). Following the incubation of logarithmic-phase bacteria (see above) for 24 h at 37°C in 96-well plates, 100 μL of phage P545 at MOIs of 0.1, 0.01, and 0.001 (phage-treated groups), or 100 μL of fresh medium (untreated group) was added to each well. The negative control group was the same as above. Biofilm degradation was evaluated by the crystal violet staining method described above. Each experiment was performed in triplicate.

### Statistical Analysis

All statistical analyses in this study were carried out using the GraphPad Prism 5 software package. Mean differences of negative control, untreated, and phage-treated groups of planktonic or biofilm assay were analyzed by Student’s *t*-test. Differences at *P* ≤ 0.05 were considered significant.

## Results

### Molecular Characteristics of the Clinical Isolates

Multilocus sequence typing showed that 54 *K. pneumoniae* belonged to seven different STs, with ST11 being the most common (48/54, 88.9%). Among 48 ST11 isolates, 24 isolates belonged to capsular K locus type KL64, 23 isolates belonged to KL47, and 1 isolate belonged to KL25. The other 6 isolates belonged to ST147, ST230, ST3155, ST3369, ST367, and ST656, and were assigned as capsular K locus type KL10, KL136, KL139, KL116, KL1, and KL30, respectively. All 54 *K. pneumoniae* isolates were carbapenemase producers, harboring *bla*_KPC–__2_ (49/54, 90.7%), *bla*_NDM–__1_ (5/54, 9.3%), and *bla*_IMP–__4_ (3/54, 5.6%). Among them, three isolates coharbored two carbapenemase genes, with two strains harboring both *bla*_KPC–__2_ and *bla*_NDM–__1_, and the other one harboring both *bla*_NDM–__1_ and *bla*_IMP–__4_. Meanwhile, 48 isolates (88.9%) were identified to carry ESBLs and/or AmpC-encoding genes. Among them, 47 isolates were found to carry *bla*_CTX–M_, including *bla*_CTX–M–__65_ (*n* = 43), *bla*_CTX–M–__3_ (*n* = 2), *bla*_CTX–M–__14_ (*n* = 1), and *bla*_CTX–M–__55_ (*n* = 1), while 16 isolates carried *bla*_SHV–__12_ and 10 isolates contained *bla*_DHA–__1_. The results are summarized in Table 1.

The result of antibiotic susceptibility testing showed 54 (100%) *K. pneumoniae* were all carbapenem resistant (Table 2). Fifty (92.6%) isolates (46 KPC-2, 1 IMP-4, and 2 NDM-1 producers and 1 KPC-2 and NDM-1 producer) exhibited minimum inhibitory concentrations (MICs) of 32 or 16 mg/L for meropenem. Fifty (92.6%) isolates (47 KPC-2, 1 NDM-1, and 2 KPC-2 and NDM-1 producers) exhibited MICsof 32 or 16 mg/L for imipenem, while the other 4 (7.4%) isolates (2 IMP-4, 1 NDM-1, and 1 IMP-4 and NDM-1 producer) exhibited MICs of 4 or 8 mg/L for imipenem (Table 2).

**TABLE 2:** Minimal inhibitory concentration of meropenem and imipenem against *Klebsiella pneumoniae*.

				**Antimicrobial**	
**Strain no.**	**Carbapenemase**	**MIC (mg/L)**		**susceptibility**	
			
		**MEM**	**IPM**	**MEM**	**IPM**
KP1	KPC-2	32	32	R	R
KP2	KPC-2	16	16	R	R
KP3	NDM-1	16	8	R	I
KP4	KPC-2	16	16	R	R
KP5	KPC-2	16	16	R	R
KP6	KPC-2	16	16	R	R
KP7	KPC-2	32	32	R	R
KP8	KPC-2	16	16	R	R
KP9	KPC-2	16	16	R	R
KP10	KPC-2	16	16	R	R
KP11	KPC-2	16	16	R	R
KP12	KPC-2	16	16	R	R
KP13	KPC-2	16	16	R	R
KP14	KPC-2	16	16	R	R
KP15	KPC-2	16	16	R	R
KP16	KPC-2	16	16	R	R
KP17	KPC-2	16	16	R	R
KP18	KPC-2	16	16	R	R
KP19	KPC-2	16	16	R	R
KP20	KPC-2	16	16	R	R
KP21	KPC-2	16	16	R	R
KP22	KPC-2	16	16	R	R
KP23	KPC-2	32	32	R	R
KP24	KPC-2	16	16	R	R
KP25	KPC-2	16	16	R	R
KP26	KPC-2	16	16	R	R
KP27	KPC-2	16	16	R	R
KP28	KPC-2	16	16	R	R
KP29	IMP-4	16	8	R	I
KP30	KPC-2	16	16	R	R
KP31	KPC-2	16	16	R	R
KP32	KPC-2	16	16	R	R
KP34	KPC-2	16	32	R	R
KP35	KPC-2	16	16	R	R
KP36	NDM-1	16	16	R	R
KP37	KPC-2	16	16	R	R
KP38	KPC-2	16	16	R	R
KP39	KPC-2	32	32	R	R
KP40	KPC-2	16	16	R	R
KP41	KPC-2	16	16	R	R
KP42	KPC-2	16	16	R	R
KP43	KPC-2	16	16	R	R
KP44	KPC-2	NA	16	NA	R
KP45	KPC-2	16	16	R	R
KP46	KPC-2	16	16	R	R
KP47	KPC-2	16	16	R	R
KP48	KPC-2	16	16	R	R
KP49	KPC-2	16	16	R	R
KP50	KPC-2	16	16	R	R
KP51	KPC-2	16	16	R	R
KP55	IMP-4; NDM-1	NA	8	NA	I
KP56	IMP-4	1	4	S	I
KP57	KPC-2; NDM-1	16	16	R	R
KP58	KPC-2; NDM-1	NA	16	NA	R

*I, intermediate; IPM, imipenem; MEM, meropenem; R, resistant; S, susceptible. NA, not available. I, intermediate; IPM, imipenem; MEM, meropenem; S, susceptible. NA, not available.*

### Phage Isolation

A total of 54 phages were isolated from two types of sewage water samples (medical sewage and domestic sewage) between July and September 2019. Among the 54 phages, 27, 14, and 8 phages were isolated from three medical sewage samples from site C, B, and A respectively, while five phages were isolated from a domestic sewage sample from site D (Supplementary Table S1).

### Host Ranges of 54 Phages

**FIGURE 1:**
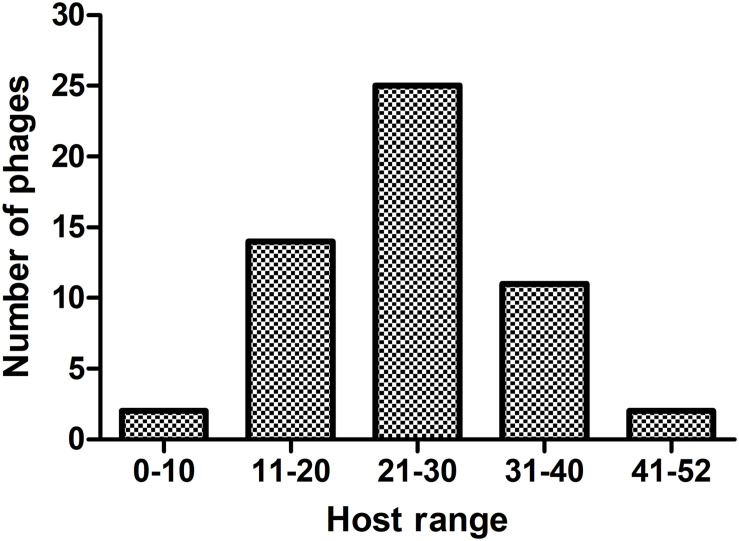
The host-range frequency distribution of phages. The *x*-axis represents five host-range groups based on the number of CRKP host strains. The *y*-axis represents the number of phages.

The 54 phages can lyse on average 25 CRKP strains, and each phage can lyse at least one ST11 strain. The host-range frequency analysis of phages showed that 2 (3.7%), 11 (20.4%), 25 (46.3%), 14 (25.9%), and 2 (3.7%) phages lysed >41, 31–40, 21–30, 11–20, and <10 CRKP strains, respectively (Figure 1).

**FIGURE 2:**
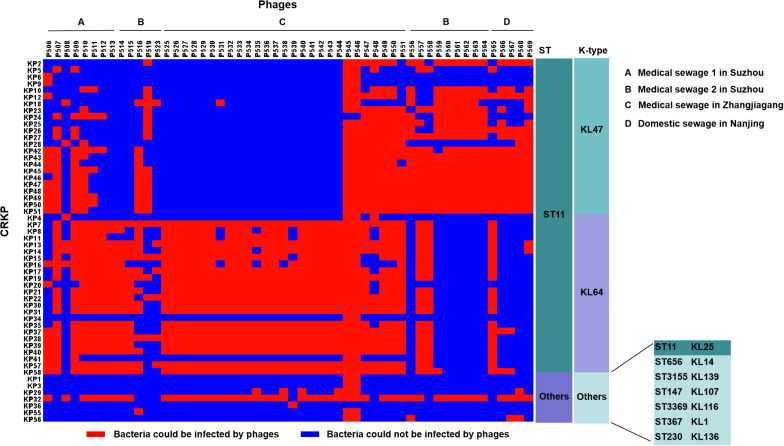
The host ranges of 54 phages. The host range was determined using 54 clinical CRKP isolates. Blue squares represent the CRKP strains (left) that cannot be infected by the corresponding phages (top). Red squares represent the CRKP strains that could be infected by corresponding phages. **(A–D)** represent different sources of sewage. **(A–C)** were from hospital and **(D)** was from domestic sewage wastewater.

Among these 54 phages, phages P545 and P546, both isolated from a sewage wastewater sample from a local hospital (site C), formed clear lysis spots on the lawns of 52 out of 54 (96.3%) CRKP strains, including both ST11 (KL47, KL64, and KL25) and non-ST11 strains, indicating that P545/P546 had a relatively wide host range (Figure 2). Two CRKP strains−KP24 (ST11, KL47) and KP36 (ST230, KL136) −were insensitive to both phage P545 and P546. By contrast, phage P523 and P508 had narrow host ranges and could form clear lysis spots on the lawns of only eight and seven strains, respectively.

The host ranges of some phages appear to be correlated with the capsular types of host bacteria. Fourteen phages (25.9%) showed an ability to lyse both ST11 KL47-type and KL64-type strains, including phages P506, P507, P509–P512, P547–P551, P557, and P558 isolated from medical sewage, and one phage P565 isolated from domestic sewage. By contrast, eight phages lysed mainly ST11 KL47 strains, including P556, P559–P564 from medical sewage, and P568 from domestic sewage (Figure 2). Twenty-three phages lysed mainly KL64-type CRKP strains, for example, P513–P515 and P525–P544, isolated mostly from medical sewage (Figure 2).

### Morphology Analysis

**FIGURE 3:**
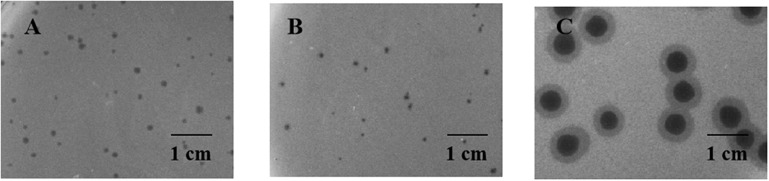
Plaque morphology of P545 **(A)**, P539 **(B)**, and P507 **(C)**. Both P545 **(A)** and P539 **(B)** formed small clear and round plaques with a diameter of approximate 0.1 cm on double layer agar plate. P507 **(C)** formed big clear and round plaques with a diameter of approximately 0.5 cm surrounded by 0.15-cm enlarged haloes.

**FIGURE 4:**
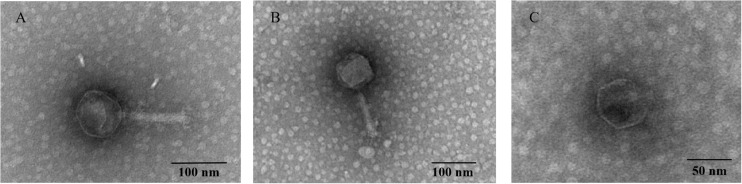
Transmission electron micrograph of phage P545 **(A)**, P539 **(B)**, and P507 **(C)**. The phage filtrate was applied to a copper grid before negative staining with phosphotungstic acid (PTA, 2% w/v). Electron micrographs were observed using an H_7650 (Hitachi, Tokyo, Japan) TEM.

Two relatively wide host-range phages, P545 and P539, and a high lytic activity phage, P507, were selected for plaque morphology analysis. Both P545 and P539 formed small clear and round plaques with a diameter of approximately 0.1 cm. P507 formed large clear and round plaques with a diameter of approximately 0.5 cm surrounded by 0.15-cm enlarged haloes, indicating that P507 most likely encodes depolymerases with polysaccharide-degrading activity (Figure 3). In addition, phages P560, P569, and P551 also formed big clear and round plaques surrounded by enlarged haloes (data not shown). TEM confirmed that P545 and P539 belonged to T4-like phages, a member of Myoviridaes, order Caudovirales. P507 belonged to T7-like phage, a member of Podoviridaes, order Caudovirales. The diameter of capsid of P507 was estimated at 50 ± 0.5 nm (Figure 4).

### Optimal MOI and One-Step Growth Curve

**FIGURE 5:**
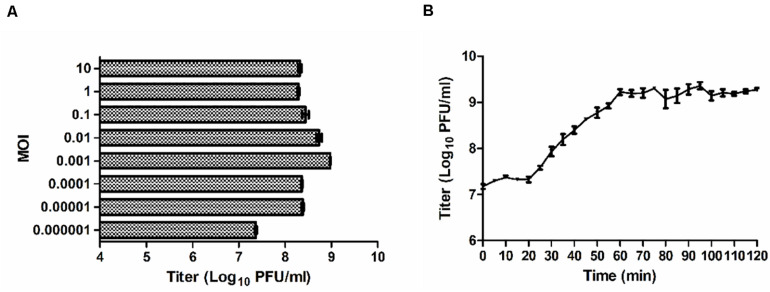
Growth characteristics of P545. **(A)** The phage titers under different MOI (phage/bacteria = 10, 1, 0.1, 0.01, 0.001, 0.0001, 0.00001, 0.000001) are indicated on the *y*-axis. **(B)** One-step growth curve test of P545 was carried out at MOI = 0.1. The results are shown as mean ± SEM from three biological replicates.

When the MOI was 0.01 or 0.001, the titers of P545 reached maximum values after propagation with a titer of approximately 10^9^ PFU/mL (Figure 5A). Considering the input–output ratio (or growth multiple) of phages, the low MOI (0.001) was considered as the optimal MOI. The one-step growth curve experiment indicated that the latent time period of phage P545 was approximately 20 min, and the burst size was about 82 phages/cell (Figure 5B).

### Thermo and pH Stability of the Phages

**FIGURE 6:**
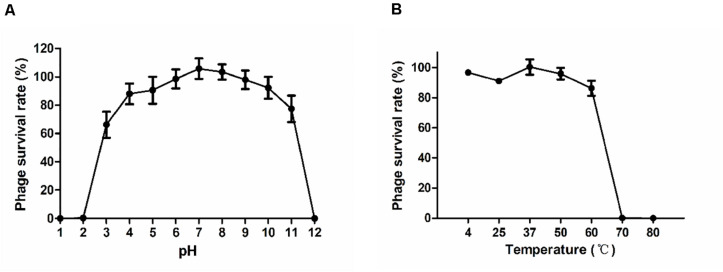
Stability tests of P545. **(A)** pH stability: phage particles were incubated under different pH conditions for 1 h. **(B)** Thermal stability: phage particles were incubated at various temperatures for 1 h. Phage survival rate = (titer after incubation)/(initial titer). SEM are shown as vertical lines.

The stability of phage P545 at various pH values and temperatures was examined. The survival rates of P545 were stable at pH 4 to pH 10 (phage survival rates >80%). However, the survival rates decreased significantly at the acidic (pH < 3) or alkaline (pH > 11) conditions (Figure 6A). After incubation for1 h, high survival rates were maintained between 4°C and 37°C (approaching 100.0%). When the temperature reached 50°C to 60°C, the survival rates declined to 95.9% and 86.2%, respectively. When the temperature reached 70°C and 80°C, nearly no phages survived (Figure 6B).

### Genome Analysis of P545

**FIGURE 7:**
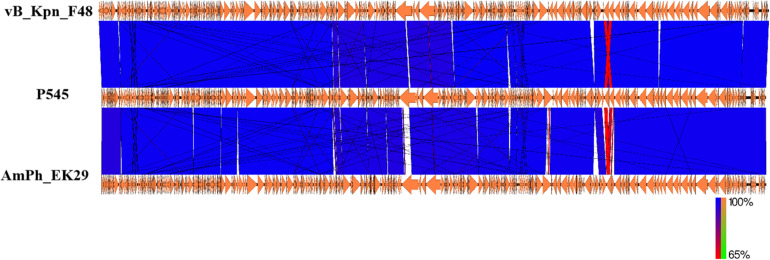
Comparative genome analysis of *K. pneumoniae* P545 bacteriophage. Schematics were produced using EasyFig. The genome sequence of the P545 was compared to those of two most closely related phages, vB_Kpn_F48 (MG746602) and AmPh_EK29 (MN434092) published in NCBI.

Next-generation sequencing analysis showed that P545 was a linear dsDNA molecule of 169,725 bp and a GC content of 40.8%. No repeated terminal sequences were detected at the 5′- and 3′-ends. P545 contained a total of 287 putative coding regions (CDSs) and 7 tRNAs. No lysogeny or virulence associated genes were identified in the genome of P545, showing its lytic nature and the potential for therapeutic application. Two putative tail lysozymes (gene loci: 7096–7590 and 157173–158912) were identified based on the RAST predication. Online BLASTn analysis of P545 genome sequence revealed that it is mostly close to *Klebsiella* phage vB_Kpn_F48 (Accession no. MG746602) isolated from a hospital sewage wastewater in Italy (Ciacci et al., 2018), with 99% query coverage and 99% nucleotide identity, and to *Klebsiella* phage AmPh_EK29 (MN434092), isolated from sewage and wastewater in Australia, with 96% query coverage and 98% nucleotide identity (Figure 7).

### Antibacterial and Antibiofilm Activity of Bacteriophage P545

**FIGURE 8:**
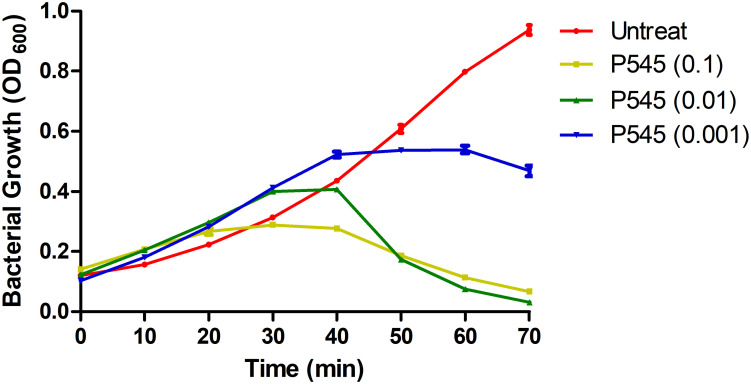
*In vitro* activity of phage P545 at 37°C. Panels show the OD_600_ values of bacterial cultures. The yellow line represents the OD_600_ value of CRKP KP4 treated with phage P545 at MOI 0.1; the green line represents that of KP4 treated with phage P545 at MOI 0.01; the blue line represents that of KP4 treated with phage P545 at MOI 0.001; the red line represents that of untreated KP4.

To evaluate the inhibitory activity of phage P545 against planktonic bacteria, P545 suspensions of different concentrations (MOI = 0.1, 0.01, and 0.001) were incubated with CRKP strain KP4 for 70 min. The antibacterial assay showed that the OD_600_ values of three phage-treated groups (MOI = 0.1, 0.01, and 0.001) were significantly lower than those of the untreated group after 50, 60, and 70 min of incubation (Figure 8). The results indicated that phage P545 can effectively inhibit bacterial growth.

**FIGURE 9:**
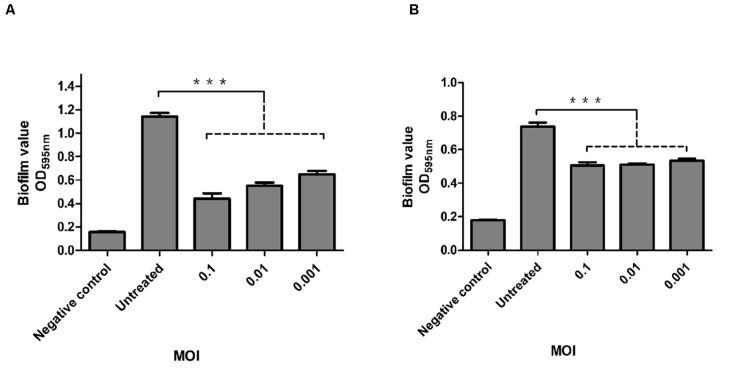
Antibacterial and antibiofilm activity of phage P545. **(A)** P545 inhibited the formation of biofilms. P545 was incubated with CRKP strain KP4 at MOIs of 0.1, 0.01, and 0.001 in 96-well plates for 24 h. **(B)** P545 disrupted the mature biofilm of KP4. Biofilm formation of KP4 was induced in 96-well plates for 24 h, and then the biofilm was treated with P545 at MOIs of 0.1, 0.01, and 0.001 for 24 h. Fresh TSB medium was used as a negative control. The OD_595_ values of the residual biofilms were measured after crystal violet staining. All the data are shown as the mean ± SEM. ***indicates *p* < 0.001.

The inhibitory effect of phage P545 on biofilm formation in the KP4 strain was analyzed using 96-well plates. To evaluate the ability of phage P545 to inhibit biofilm formation, phage P545 suspensions were incubated with CRKP strain KP4 at different MOIs (0.1, 0.01, and 0.001) for 24 h. The results of crystal violet staining assay showed that the OD_595_ values of three different phage-treated groups (MOI 0.1, 0.01, and 0.001) decreased 0.70, 0.59, and 0.49, respectively, in comparison to that of the untreated group (Figure 9A, *P* < 0.001). The ability of P545 to degrade biofilms was also assessed using 24-h-old mature biofilms. The OD_595_ values of the total biomass that remained attached in the phage-treated groups decreased 0.24 (MOI = 0.1), 0.23 (MOI = 0.01), and 0.21 (MOI = 0.001) compared with that of the untreated group (Figure 9B, *P* < 0.001). These results suggested that P545 can inhibit biofilm formation and degrade the formed mature biofilm.

## Discussion

Carbapenem-resistant *Klebsiella pneumoniae* strains have emerged as significant multidrug-resistant bacterial pathogens that pose a serious threat to global public health. The international spreads of CRKP strains were associated with a few high-risk clonal strains, including ST258, ST11, ST14, ST147, and ST307 etc. Among them, ST258 predominates in North America and Europe, whereas ST11 is the primary CRKP clone in East Asia, especially China (Chen et al., 2014; Zhang et al., 2019). In China, ST11 strains accounted for >70% CRKP strains (Liu et al., 2018). CRKP ST11 strains were primarily associated with the *bla*_KPC–__2_ gene in China, but other carbapenemase genes, such as *bla*_NDM_, *bla*_OXA–__48__–like_, *bla*_VIM_, and *bla*_IMP_, were frequently identified in ST11 strains elsewhere (Chen et al., 2018; Liu et al., 2018; Wang et al., 2018). For example, in this study, two ST11 strains were also found to carry both *bla*_NDM–__1_ and *bla*_KPC–__2_. In addition, some CRKP ST11 strains have evolved to be extensively drug resistant (Dorp et al., 2019; Wu X. et al., 2019). ccordingly, combating the infection caused by epidemic CRKP ST11 strains has already become a priority in controlling CRKP strains in China and some other countries.

Bacteriophages are being considered as a potential therapeutic approach against highly drug-resistant pathogens, including CRKP strains (Wu X. et al., 2019). In this study, we isolated 54 phages from domestic and hospital sewage wastewater. Our results suggested that sewage, especially medical sewage, may be a better phage source against clinical CRKP strains, since phages can be efficiently isolated (Peng et al., 2020). Clinically, phages with relatively wide host ranges could be ideal candidates for phage cocktail in phage therapy. Based on the host-range analysis, three selected phages (i.e., P545, P539, and P507) can potentially cover all 54 clinical CRKP isolates, collected from various clinical sources and harboring different carbapenemase genes.

Our results also indicated that the phage lytic activity was independent of the carbapenemase genotypes. Importantly, all 54 phages showed lytic activity against some epidemic ST11 strains. The host ranges of certain phages appeared to be narrow and were specific to certain *K. pneumoniae* strains. This result was similar to the results of other studies that some phages were specific to certain capsular serotypes. For example, bacteriophage NTUH-K2044-K1-1 isolated from Taiwan exhibited specificity for capsular type K1 (Lin et al., 2014). *Klebsiella* bacteriophage K5-2 exhibited specificity for capsular types K5, K30, and K69, and bacteriophage K5-4 exhibited specificity for capsular types K8 and K5 (Hsieh et al., 2017). Three bacteriophages−KpV41, KpV475, and KpV71−had specific lytic activity against mainly K1 stains (Solovieva et al., 2018). Moreover, two phages, KpV74, and KpV763, had specific lytic activity against K2 strains, and the phage KpV767 had specific lytic activity against K57 strains (Solovieva et al., 2018).

Interestingly, we identified a phage, P545, with a relatively wide host range, that can lyse 52 of 54 (96.3%) CRKP strains from our collection. These 52 CRKP strains included 24 ST11 KL64, 22 ST11 KL47, 1 ST11 KL25, 1 ST367 KL1, 1 ST147 KL10, 1 ST656 KL14, 1 ST3155 KL139, and 1 ST230 KL136. The molecular mechanism underlying the relatively wide host range of phage P545 remains unclear. TEM of phage P545 revealed that P545 belonged to T4-like lytic phage, a member of Myoviridaes, order Caudovirales. BLASTn analysis showed that P545 was highly similar to *Klebsiella* phage vB_Kpn_F48 (MG746602) from Italy and AmPh_EK29 (MN434092) from Australia. However, both vB_Kpn_F48 and AmPh_EK29 appear to be narrow host-range phages. Phage vB_Kpn_F48 had a lytic activity specific to *K. pneumoniae* strains belonging to ST101 (K17) and its closely related strains (Ciacci et al., 2018). Similarly, AmPh_EK29 was specifically active against *K. pneumoniae* ST258 clade 1 strains (KL106). Our phage P545 had a lytic activity against ST11 strains (KL47 and KL64) and six other STs. Further studies will be performed to examine whether P545 can actively lyse other CRKP strains, especially ST258 and ST101 strains. Our genome alignment results showed that P545 harbors >2600 and >4600 core single nucleotide polymorphisms and some insertion or deletion versus vB_Kpn_F48 and AmPh_EK29, respectively. For T4-like phages, the tail fiber protein gene in general determined the specificity and host range. BLASTn analysis revealed the tail fiber protein gene sequence of P545 had only 91% query coverage and 89.4% nucleotide identity (genome level: 99% query coverage and 99% nucleotide identity) with that of *Klebsiella* phage vB_Kpn_F48. We suspected the genetic variation in tail fiber protein gene of P545, in comparison to vB_Kpn_F48 and AmPh_EK29, may contribute to the relatively wide host range observed in P545, which deserves further study.

Phage P545 had a short latent period of 20 min and a medium burst size of 82 PFU/cell and was stable at pH values (4–10) and temperatures (up to 60°C). When the MOI was 0.01 or 0.001, the titers of P545 reached the maximum value (no significant difference). Considering the input–output ratio (or growth multiple) of phage, the lower MOI (0.001) was considered the optimal MOI.

In this study, phage P545 inhibited bacterial growth within 70 min. The result of crystal violet staining assay suggested that phage P545 can not only inhibit biofilm formation but also degrade the formed mature biofilms. Many bacteriophages produced specific depolymerases, which are able to destroy the capsular polysaccharides, thereby allowing adsorption of phages to the outer membrane receptors (Pires et al., 2016) and penetration of phage DNA into the bacterial cells (Hughes et al., 1998). These phage-derived capsule depolymerases can be potentially used as therapeutic agents against capsulated bacteria, such as CRKP strains. However, the plaque morphology analysis suggested that phage P545 was a non-depolymerase-producing phage, because no clear enlarged haloes were observed (Figure 4). In fact, besides depolymerases, phage-encoded lysozymes (endolysins, lysins, etc.) can also digest bacterial cell wall material (Chan and Abedon, 2015; Ajuebor et al., 2016). Two tail lysozyme genes were found in the genome of P545 based on RAST predication. Therefore, we suspected that the mechanism of biofilm formation inhibition and biofilm degradation may be associated with phage-encoded tail lysozymes of phage P545. The results suggested that P545 could be an attractive candidate as an anti-CRKP agent. Moreover, in this study, P507 formed large clear and round plaques surrounded by enlarged haloes, suggesting that it most likely encodes depolymerases with polysaccharide-degrading activity (Pan et al., 2017; Wu X. et al., 2019).

One limitation of current study was that we determined the phage activities against CRKP isolates collected from only a single medical center. Nevertheless, these CRKP isolates were collected from various clinical sources and harbored a wide battery of carbapenemase genes, including the most predominant STs and capsular types of strains spreading in China. In addition, another limitation is that only one of the 54 phages (P545) was fully characterized. Further work will be conducted to characterize other phages isolated in the current study, such as P507, and to test the activities of these phages against CRKP isolates from other genetic backgrounds. Today, the sources and rapid availability of phages against clinically isolated pathogens remains the most significant challenge for phage therapy. Our study described that the 54 phages can be successfully obtained within a short period of time (1.5 months) from local sewage, especially from medical wastewater. Medical sewage in the surrounding area could serve as a rich naturally occurring phage source against clinically important pathogens. Natural phage, in combination with engineered phages and phage-encoded enzymes, may provide a more effective solution for combating epidemic CRKP infections.

## Data Availability Statement

The datasets generated for this study can be found in the annotated whole-genome sequence of phage P545 was deposited in the GenBank database under accession number: MN781108.

## Ethics Statement

This study was approved by the Institutional Review Board (IRB) of The Second Affiliated Hospital of Soochow University. The clinical isolates were retrospectively collected, and patient data were not included in this study, therefore informed consent was waived by the Institutional Review Board (IRB) of The Second Affiliated Hospital of Soochow University.

## Author Contributions

ML, WZ, and HD conceived the study. ML, MG, LoC, PL, YX, and WZ performed the experiment and computational analysis. ML, LiC, HG, and WZ wrote the article. All authors read, revised, and approved the final manuscript.

## Conflict of Interest

The authors declare that the research was conducted in the absence of any commercial or financial relationships that could be construed as a potential conflict of interest.

## Footnotes

1 https://bigsdb.pasteur.fr/klebsiella/klebsiella.html

2 https://talk.ictvonline.org/taxonomy/

3 https://blast.ncbi.nlm.nih.gov/Blast.cgi
