# Supplementary material for: Isolation and Characterization of Novel Lytic Bacteriophages Infecting Epidemic Carbapenem-Resistant Klebsiella pneumoniae Strains
Source: Front Microbiol. 2020 Jul 21;11:1554. doi: 10.3389/fmicb.2020.01554 (PMC7385232; doi:10.3389/fmicb.2020.01554)
Supplement: Supplementary file 1 [file Data_Sheet_1.PDF]

Table S1 Background information of 54 phages isolated from different sewage water

Phages	Original host strains	Host Strains Sum	Sources	Sites	Dates
P506	KP9	22	Medical sewage	Site A	2019.07.12
P507	KP17	35	Medical sewage	Site A	2019.07.12
P508	KP18	7	Medical sewage	Site A	2019.07.12
P509	KP30	35	Medical sewage	Site A	2019.07.12
P510	KP31	35	Medical sewage	Site A	2019.07.12
P511	KP35	28	Medical sewage	Site A	2019.07.12
P512	KP38	24	Medical sewage	Site A	2019.07.12
P513	KP39	21	Medical sewage	Site A	2019.09.06
P514	KP7	21	Medical sewage	Site B	2019.09.06
P515	KP3	19	Medical sewage	Site B	2019.09.06
P516	KP64	27	Medical sewage	Site B	2019.09.06
P519	KP43	21	Medical sewage	Site B	2019.09.15
P523	KP18	8	Medical sewage	Site B	2019.09.15
P525	KP8	22	Medical sewage	Site C	2019.09.19
P526	KP11	22	Medical sewage	Site C	2019.09.19
P527	KP14	22	Medical sewage	Site C	2019.09.19
P528	KP15	22	Medical sewage	Site C	2019.09.19
P529	KP16	22	Medical sewage	Site C	2019.09.19
P530	KP17	22	Medical sewage	Site C	2019.09.19
P531	KP18	20	Medical sewage	Site C	2019.09.19
P532	KP20	22	Medical sewage	Site C	2019.09.19
P533	KP21	22	Medical sewage	Site C	2019.09.19
P534	KP22	22	Medical sewage	Site C	2019.09.19
P535	KP29	20	Medical sewage	Site C	2019.09.19
P536	KP30	22	Medical sewage	Site C	2019.09.19
P537	KP31	22	Medical sewage	Site C	2019.09.19
P538	KP35	22	Medical sewage	Site C	2019.09.19
P539	KP36	19	Medical sewage	Site C	2019.09.19
P540	KP37	23	Medical sewage	Site C	2019.09.19
P541	KP38	22	Medical sewage	Site C	2019.09.19
P542	KP40	22	Medical sewage	Site C	2019.09.19
P543	KP41	22	Medical sewage	Site C	2019.09.19
P544	KP57	23	Medical sewage	Site C	2019.09.19
P545	KP4	52	Medical sewage	Site C	2019.09.19
P546	KP5	52	Medical sewage	Site C	2019.09.19
P547	KP10	38	Medical sewage	Site C	2019.09.19
P548	KP12	34	Medical sewage	Site C	2019.09.19
P549	KP49	40	Medical sewage	Site C	2019.09.19
P550	KP50	40	Medical sewage	Site C	2019.09.19
P551	KP58	37	Medical sewage	Site C	2019.09.19
P556	KP2	20	Medical sewage	Site B	2019.09.27
P557	KP13	37	Medical sewage	Site B	2019.09.27
P558	KP19	34	Medical sewage	Site B	2019.09.27
P559	KP26	20	Medical sewage	Site B	2019.09.27
P560	KP42	20	Medical sewage	Site B	2019.09.27
P561	KP47	20	Medical sewage	Site B	2019.09.27
P562	KP48	20	Medical sewage	Site B	2019.09.27
P563	KP53	20	Medical sewage	Site B	2019.09.27
P564	KP54	19	Medical sewage	Site B	2019.09.27
P565	KP19	34	Domestic sewage	Site D	2019.09.27
P566	KP24	20	Domestic sewage	Site D	2019.09.27
P567	KP26	19	Domestic sewage	Site D	2019.09.27
P568	KP28	18	Domestic sewage	Site D	2019.09.27
P569	KP51	22	Domestic sewage	Site D	2019.09.27
